# Development and validation of a nomogram-based risk prediction model for unfavorable outcomes in pediatric traumatic brain injury: a retrospective study

**DOI:** 10.3389/fped.2025.1578679

**Published:** 2025-04-11

**Authors:** Dehong Fan, Meiling Yang, Yuyan He, Xuebing Lan, Dou Lin, Wen Zhou, Yonghua Lin, Yuhui Chen, Qi Li, Jinrun Lin

**Affiliations:** ^1^ Department of Neurosurgery, Fujian Children’s Hospital (Fujian Branch of Shanghai Children’s Medical Center), College of Clinical Medicine for Obstetrics & Gynecology and Pediatrics, Fujian Medical University, Fuzhou, Fujian, China; ^2^ Department of Neurosurgery, the 900th Hospital, Fuzhou, Fujian, China; ^3^ Department of Neurosurgery, Fuzong Clinical College of Fujian Medical University, Fuzhou, Fujian, China

**Keywords:** pediatric traumatic brain injury, unfavorable outcome, risk factors, predictive model, nomogram

## Abstract

**Introduction:**

Pediatric traumatic brain injury (PTBI) is linked to significant disability and mortality. This study aimed to identify risk factors for unfavorable outcomes in patients with PTBI and develop a predictive risk model.

**Methods:**

A retrospective analysis was conducted on patients with PTBI treated at the 900th Hospital from September 2021 to June 2023. Univariate and multivariate regression analyses identified risk factors for adverse outcomes and facilitated the creation of a nomogram. The model's predictive accuracy was assessed using Receiver Operating Characteristic (ROC) curves, calibration curves, and Decision Curve Analysis (DCA). External validation was performed with patients with PTBI from Fujian Children's Hospital.

**Results:**

Key findings indicated that a Glasgow Coma Scale (GCS) score ≤8, subdural hematoma, subarachnoid hemorrhage, and coagulopathy were independent risk factors. The nomogram achieved an area under the ROC curve of 0.947 in the development cohort and 0.834 in the external validation cohort, demonstrating a good fit. DCA results confirmed that the nomogram enhanced the prediction of unfavorable outcomes.

**Conclusions:**

This risk prediction model offers high accuracy for early identification of adverse outcomes, enabling timely interventions to improve the quality of life for patients with PTBI.

## Introduction

Traumatic brain injury (TBI) is a prevalent health condition worldwide with high mortality and disability rates. Globally, approximately 69 million cases of TBI occur each year and its incidence has increased in recent years (1, 2). With the development of the economy and transportation, the incidence of pediatric traumatic brain injury (PTBI) has also been steadily rising due to characteristics such as strong curiosity and weak awareness of danger (3, 4). PTBI ranks among the top causes of traumatic death and disability in children, imposing a significant burden on low- and middle-income families (5, 6). TBI often leads to contusions, hemorrhages, and damage to neurons and axons, resulting in acute injuries and multiple complications with a poor prognosis (7). Although advancements in medical care have increased the survival rate of patients with PTBI, they still face short-term risks of physical, cognitive, emotional, and social impairments in the short term (8). Moreover, PTBI may have long-term adverse effects on development and brain maturity as children age (9, 10).

Although extensive research has been conducted on adverse prognostic factors in adult patients with TBI, studies on PTBI remained limited (11). Some identified risk factors include low Glasgow Coma Scale (GCS) scores, abnormal pupillary light reflexes, and hematological abnormalities, highlighting the crucial importance of preoperative prognosis prediction for patients with PTBI in clinical decision-making (12–14). Therefore, there was an urgent need to identify patients with PTBI at risk of an unfavorable outcome early and implement timely interventions to improve outcomes. However, there was a lack of simple, clinically applicable risk prediction models to guide the outcomes of PTBI. Early identification of PTBI and achieving favorable outcomes in subsequent medical and care decisions were crucial for healthcare professionals and the families of patients.

Therefore, this study aimed to analyze risk factors for unfavorable outcomes using routine clinical, imaging, and laboratory indicators, and to develop and validate a risk prediction model for predicting unfavorable outcomes in patients with PTBI.

## Materials and methods

### Patient population

This study includes two independent cohorts. The development cohort consists of patients with PTBI treated in the Neurosurgery Department of the 900th Hospital between September 1, 2021, and June 30, 2023. The external validation cohort comprises patients with PTBI treated in the Neurosurgery Department of Fujian Children's Hospital between January 1, 2022, and June 30, 2023. This study was approved by the Ethics Committee of Fujian Children's Hospital and was conducted according to the principles of the Declaration of Helsinki (approval number: 2024ETKLRK07003). The 900th Hospital was informed and agreed with the study. Owing to the retrospective nature of the study, the committee waived the requirement for obtaining informed consent.

The inclusion criteria were as follows: (1) age between 0 and 16 years; (2) clear history of acute TBI; (3) isolated TBI without concomitant injury to other parts of the body; and (4) admission within 12 h of injury and completion of imaging and hematological examinations within 1 h of admission.

The exclusion criteria were as follows: (1) death upon admission, defined as patients who were declared dead upon hospital arrival before undergoing any imaging or hematological examinations; (2) history of life-threatening diseases, such as malignancies, severe cardiopulmonary dysfunction, or congenital deformities; (3) presence of acute or chronic infections; (4) incomplete clinical data; and (5) incomplete follow-up information.

### Data collection

The collected data included records of the patients', age, initial GCS score upon admission, mechanism of injury, results of initial radiological examinations [such as skull base fracture, epidural hematoma (EDH), subdural hematoma (SDH), subarachnoid hemorrhage (SAH), cerebral contusion (CC), intraventricular hemorrhage (IVH)], and routine laboratory parameters [hemoglobin (Hb), albumin (Alb), C-reactive protein (CRP), international normalized ratio (INR), activated partial thromboplastin time (APTT), platelet count (PLT)].

### Definition of coagulopathy

Due to the lack of a unified international standard for coagulopathy following PTBI, we defined coagulopathy as meeting at least one of the following criteria: INR > 1.2, APTT > 35 s, PLT < 100  ×  10^3^/ml, according to previous studies (15–17).

### Outcome assessment

All patients were regularly followed up by professionals through outpatient visits, text messages, and phone calls. Patient Outcome was evaluated using the Glasgow Outcome Scale (GOS) 6 months after discharge. A GOS score of 1 indicates death; 2 indicates a vegetative state; 3 indicates severe disability and inability to live independently; 4 indicates moderate disability but ability to live with assistance; and 5 indicates good recovery and ability to resume normal life. According to the GOS score, patients were divided into a favorable outcome group (GOS score of 4–5) and an unfavorable outcome group (GOS score of 1–3).

### Statistical analysis

Statistical analyses were performed using R software version 4.2.2 (R Foundation for Statistical Computing, Vienna, Austria). The normality of continuous variables was assessed using the Shapiro–Wilk test. For normally distributed continuous data, mean ± standard deviation was used, and intergroup comparisons were made using the *t*-test. For non-normally distributed continuous data, the median and interquartile range were used, and intergroup comparisons were made using the Mann–Whitney *U* test. Categorical data are presented as percentages (*n*, %), and intergroup comparisons were performed using the chi-square test or Fisher's exact test. Factors with *P* < 0.05 in univariate analysis were included in multivariate logistic regression analysis using stepwise regression to assess independent risk factors for unfavorable outcomes in patients with PTBI. Additionally, a nomogram was constructed to visualize the risk prediction model. The diagnostic value and clinical utility of the risk prediction model were assessed using the area under the curve (AUC) of the receiver operating characteristic (ROC), calibration curves, and Decision Curve Analysis (DCA). Statistical significance was set at *P* < 0.05.

## Results

### Patient selection

**Figure 1:**
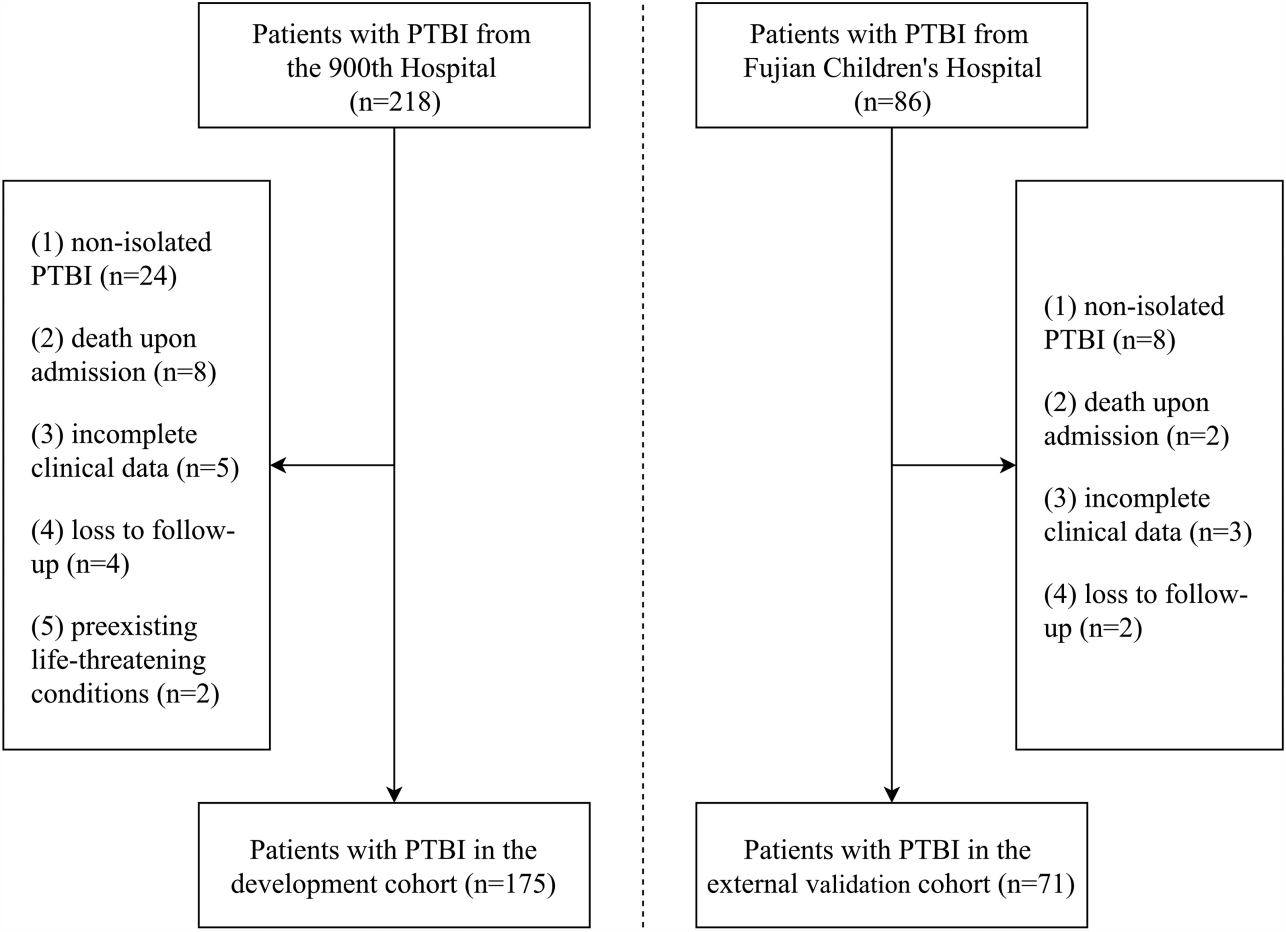
Flowchart of the selection process for patients with PTBI in the development and external validation cohorts.

**Figure 2:**
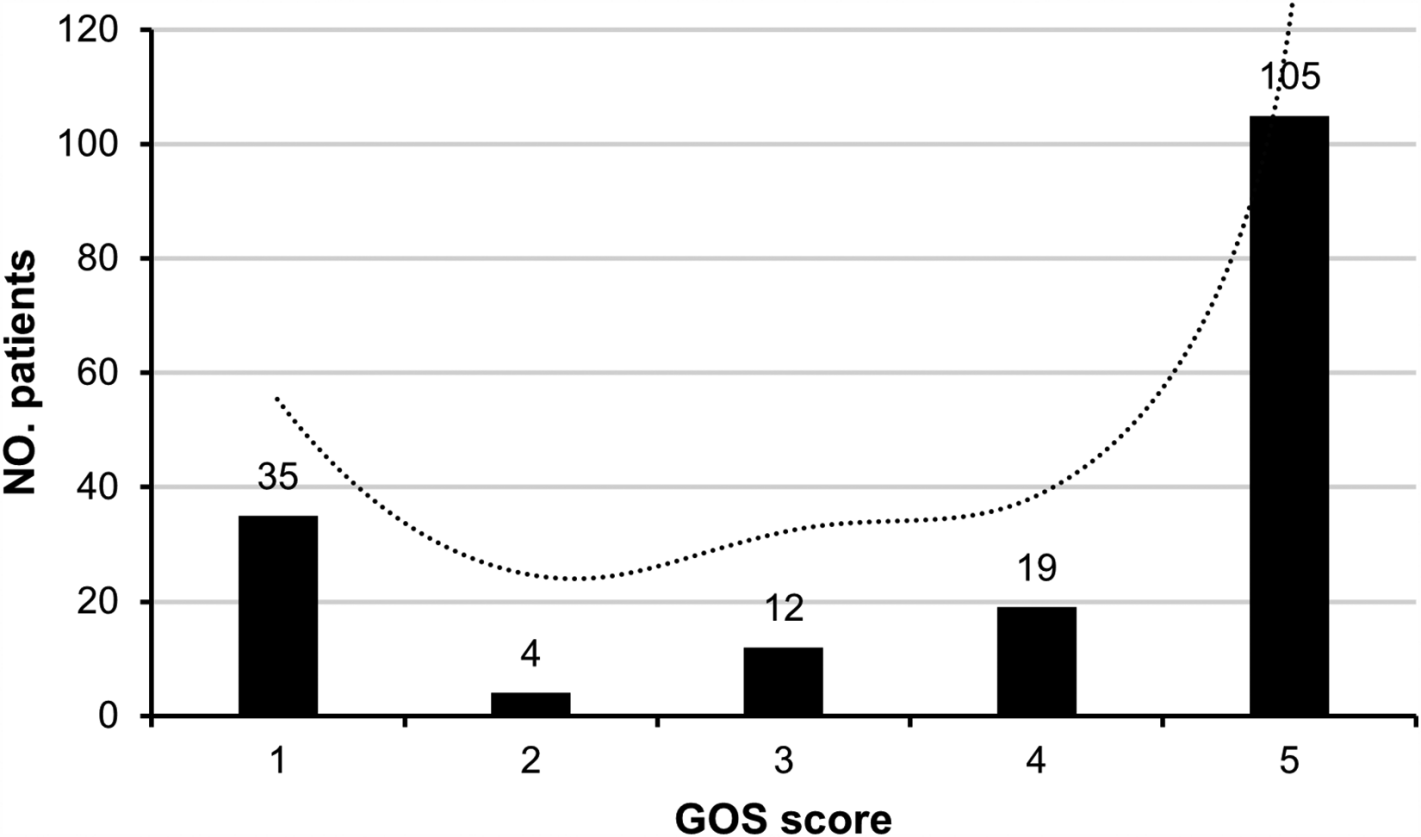
Distribution of patients according to the Glasgow outcome scale (GOS) scores.

We initially collected data from 218 patients with PTBI. After applying the inclusion and exclusion criteria, 175 patients with PTBI were included in the development cohort, and 71 patients with PTBI were included in the external validation cohort. The patient selection process is summarized in Figure 1. Based on the GOS scores at 6 months from discharge, 124 patients (70.9%) were classified into the favorable outcome group and 51 patients (29.1%) into the unfavorable outcome group. Specifically, 35, 4, 12, 19, and 105 patients had GOS scores of 1, 2, 3, 4, and 5, respectively, indicating a bimodal distribution of outcome among patients with PTBI (Figure 2).

### Characteristics of patients with PTBI in the development and external validation cohorts

Table 1 shows a comparison of characteristics between the development and external validation cohorts, and no significant differences were observed in the baseline characteristics between the two cohorts (*P* > 0.05).

**Table 1:** Comparison of the characteristics between the development and external validation cohorts.

Variable	Development cohort	Validation cohort	*P* value
	(*n* = 175)	(*n* = 71)	
Sex (%)			0.530
Male	116 (66.29)	50 (70.42)	
Female	59 (33.71)	21 (29.58)	
Age (IQR)	4.00 (1.00, 8.50)	3.00 (1.00, 6.00)	0.540
GCS ≤ 8	59 (33.71)	28 (39.44)	0.395
Mechanism of injury (%)			0.439
Traffic accident	62 (35.43)	21 (29.58)	
Fall	64 (36.57)	23 (32.39)	
Free fall	26 (14.86)	16 (22.54)	
Blow injury	23 (13.14)	11 (15.49)	
Skull base fracture (%)	94 (53.71)	31 (43.66)	0.153
EDH (%)	101 (57.71)	34 (47.89)	0.160
SDH (%)	101 (57.71)	37 (52.11)	0.422
SAH (%)	91 (52.00)	38 (53.52)	0.829
CC (%)	79 (45.14)	30 (42.25)	0.679
IVH (%)	9 (5.14)	6 (8.45)	0.491
Coagulopathy (%)	41 (23.43)	21 (29.58)	0.314
Hb (IQR)	122.00 (108.00, 129.50)	118.00 (107.00, 125.00)	0.136
Alb (IQR)	43.20 (39.65, 45.70)	41.00 (38.80, 44.85)	0.118
CRP (IQR)	1.00 (0.80, 3.00)	1.20 (1.00, 2.10)	0.136
PLT (IQR)	205.00 (153.50, 239.00)	190.00 (157.00, 218.50)	0.286
INR (IQR)	1.07 (0.99, 1.17)	1.04 (0.98, 1.12)	0.135
APTT (IQR)	26.80 (24.90, 30.50)	28.10 (25.60, 32.40)	0.189
Unfavorable outcome (%)	51 (29.14)	26 (36.62)	0.252

IQR, interquartile range; GCS, Glasgow coma scale; EDH, epidural hematoma; SDH, subdural hematoma; SAH, subarachnoid hemorrhage; CC, cerebral contusion; IVH, intraventricular hemorrhage; Hb, hemoglobin; Alb, albumin; CRP, C-reactive protein; PLT, platelet count; INR, international normalized ratio; APTT, activated partial thromboplastin time.

### Baseline characteristics of patients with PTBI in the development cohort

**Figure 3:**
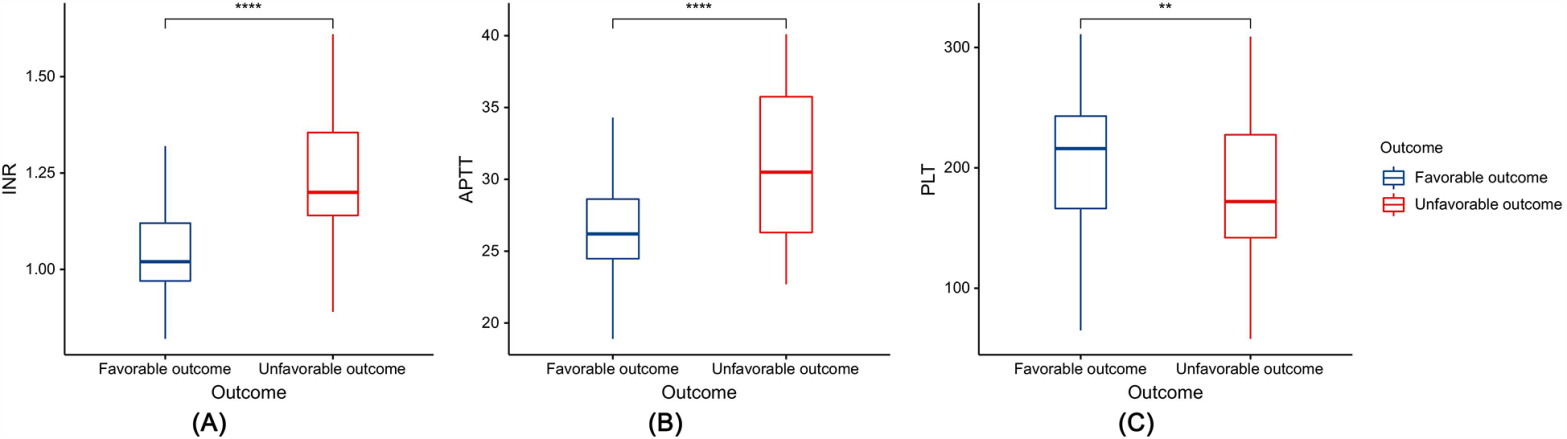
Box plots depicting the relationship between coagulation indicators and prognosis: **(A)** international normalized ratio (INR), **(B)** activated partial thromboplastin time (APTT), and **(C)** platelet count (PLT). **P < 0.01, ****P < 0.0001.

Among the 175 patients with PTBI included in the development cohort, there were 116 male (66.29%) and 59 female patients (33.71%), with a median age of 4.00 (1.00–8.50) years. The results of the univariate analysis showed that a GCS score at admission ≤8, SDH, SAH, CC, IVH, and coagulopathy were risk factors for unfavorable outcomes in patients with PTBI (*P* < 0.05). However, sex, age, mechanism of injury, skull base fracture, and EDH, Hb, Alb, and CRP levels were not associated with unfavorable outcomes in patients with PTBI (*P* > 0.05) (Table 2). Additionally, compared with the patients with PTBI with a favorable outcome, those with an unfavorable outcome had significantly elevated INR (1.02 vs. 1.20, *P* < 0.0001) and APTT (26.20 vs. 30.50, *P* < 0.0001) and significantly decreased PLT (216.00 vs. 172.00, *P* < 0.01) (Figure 3).

**Table 2:** Differences between pediatric traumatic brain injury with favorable and unfavorable outcomes in the development cohort.

Variable	Favorable outcome	Unfavourable outcome	*P* value
	(*n* = 124)	(*n* = 51)	
Sex (%)			0.674
Male	81 (65.32)	35 (31.37)	
Female	43 (34.68)	16 (68.63)	
Age (IQR)	4.00 (1.00, 8.00)	3.00 (1.00, 9.00)	0.954
GCS ≤ 8	18 (14.52)	41 (80.39)	<0.001
Mechanism of injury (%)			0.311
Traffic accident	46 (37.10)	16 (31.37)	
Fall	40 (32.26)	24 (47.06)	
Free fall	20 (16.13)	6 (11.76)	
Blow injury	18 (14.52)	5 (9.80)	
Skull base fracture (%)	65 (52.42)	29 (56.86)	0.592
EDH (%)	76 (61.29)	25 (49.02)	0.135
SDH (%)	62 (50.00)	39 (76.47)	0.001
SAH (%)	43 (34.68)	48 (94.12)	<0.001
CC (%)	47 (37.90)	32 (62.75)	0.003
IVH (%)	3 (2.42)	6 (11.76)	0.030
Coagulopathy (%)	13 (10.48)	28 (54.90)	<0.001
Hb (IQR)	122.00 (108.00, 128.25)	120.00 (108.00, 130.00)	0.933
Alb (IQR)	43.15 (39.70, 45.52)	43.80 (39.30, 46.10)	0.894
CRP (IQR)	0.90 (0.80, 3.70)	1.00 (1.00, 2.00)	0.193

IQR, interquartile range; GCS, Glasgow coma scale; EDH, epidural hematoma; SDH, subdural hematoma; SAH, subarachnoid hemorrhage; CC, cerebral contusion; IVH, intraventricular hemorrhage; Hb, hemoglobin; Alb, albumin; CRP, C-reactive protein.

### Development of a risk prediction model for unfavorable outcomes in PTBI

**Figure 4:**
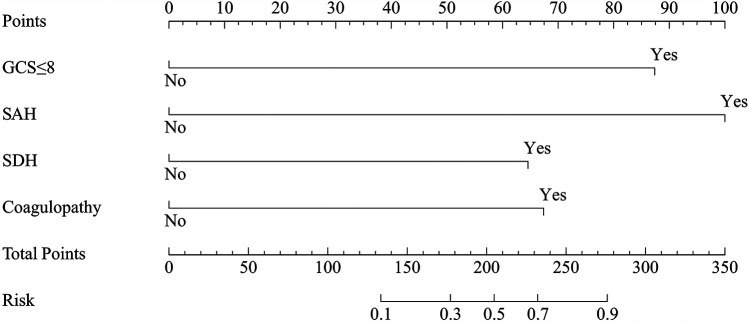
The nomogram for predicting unfavorable outcomes in pediatric traumatic brain injury. Prediction points can be found on the highest point scale corresponding to each patient variable and can be summed. The total score, projected onto the bottom scale, represents the risk of developing unfavorable outcomes. GCS, Glasgow coma scale; SAH, subarachnoid hemorrhage; SDH, subdural hematoma.

Risk factors with a *P* value of <0.05 in the univariate analysis were included in the multivariate logistic regression analysis. This analysis identified a GCS score ≤8, SAH, SDH, and coagulopathy as independent predictors of unfavorable outcomes in patients with PTBI (Table 3). A risk prediction model based on these independent predictors was subsequently developed and illustrated using a nomogram (Figure 4).

**Table 3:** Multivariate logistic regression analysis of unfavorable outcomes in 175 patients with pediatric traumatic brain injury.

Variable	OR	95% CI	*P* Value
GCS ≤ 8	14.794	4.782–45.768	<0.001
SAH	21.830	4.743–100.482	<0.001
SDH	7.323	2.030–26.422	0.002
Coagulopathy	7.987	2.305–27.681	0.001

CI, confidence interval; GCS, Glasgow coma scale; SAH, subarachnoid hemorrhage; SDH, subdural hematoma.

### External validation of the nomogram

**Figure 5:**
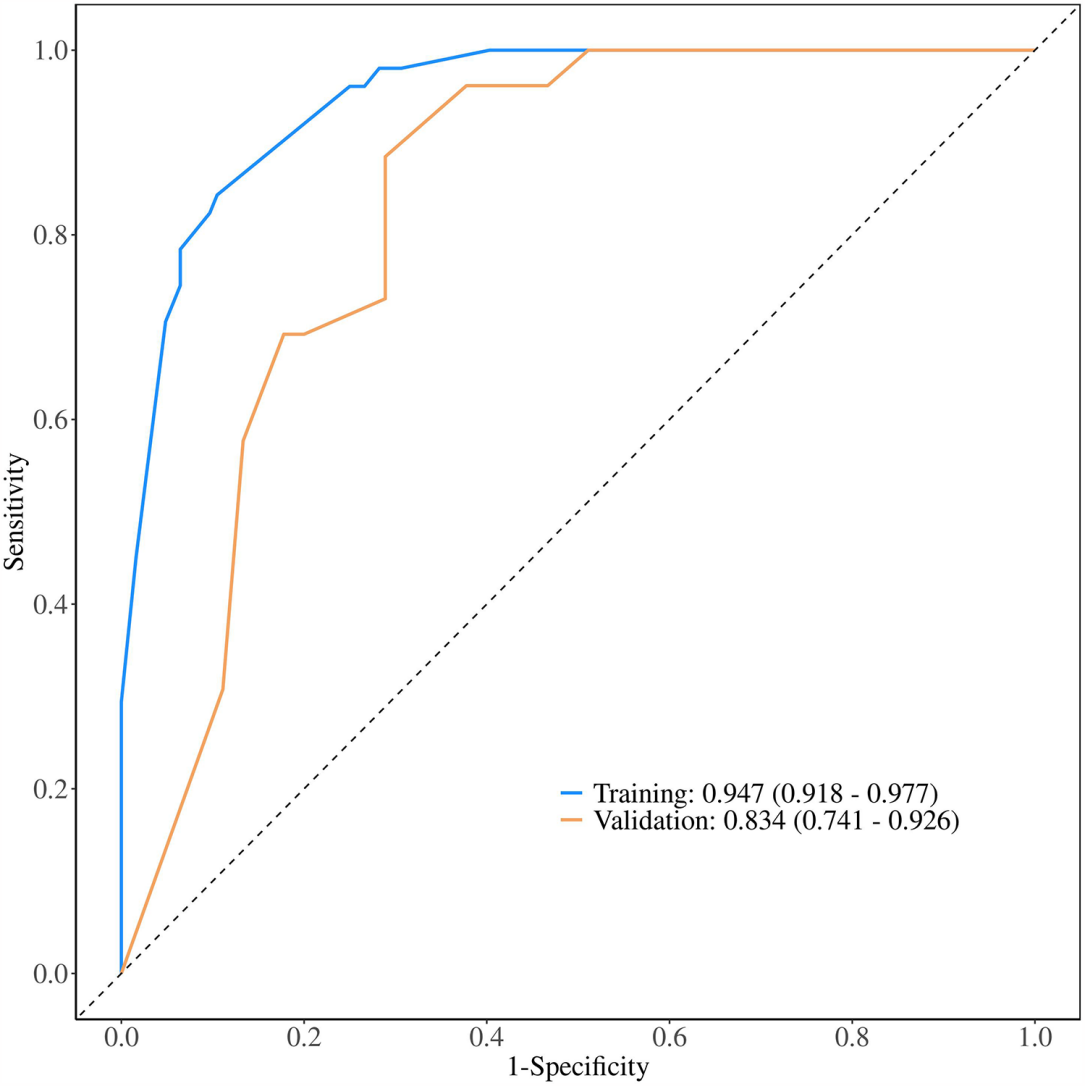
Receiver operating characteristic curve of the nomogram. The nomogram demonstrates good discriminative ability, with an area under the ROC curve of 0.947 in the development cohort and 0.834 in the external validation cohort.

**Figure 6:**
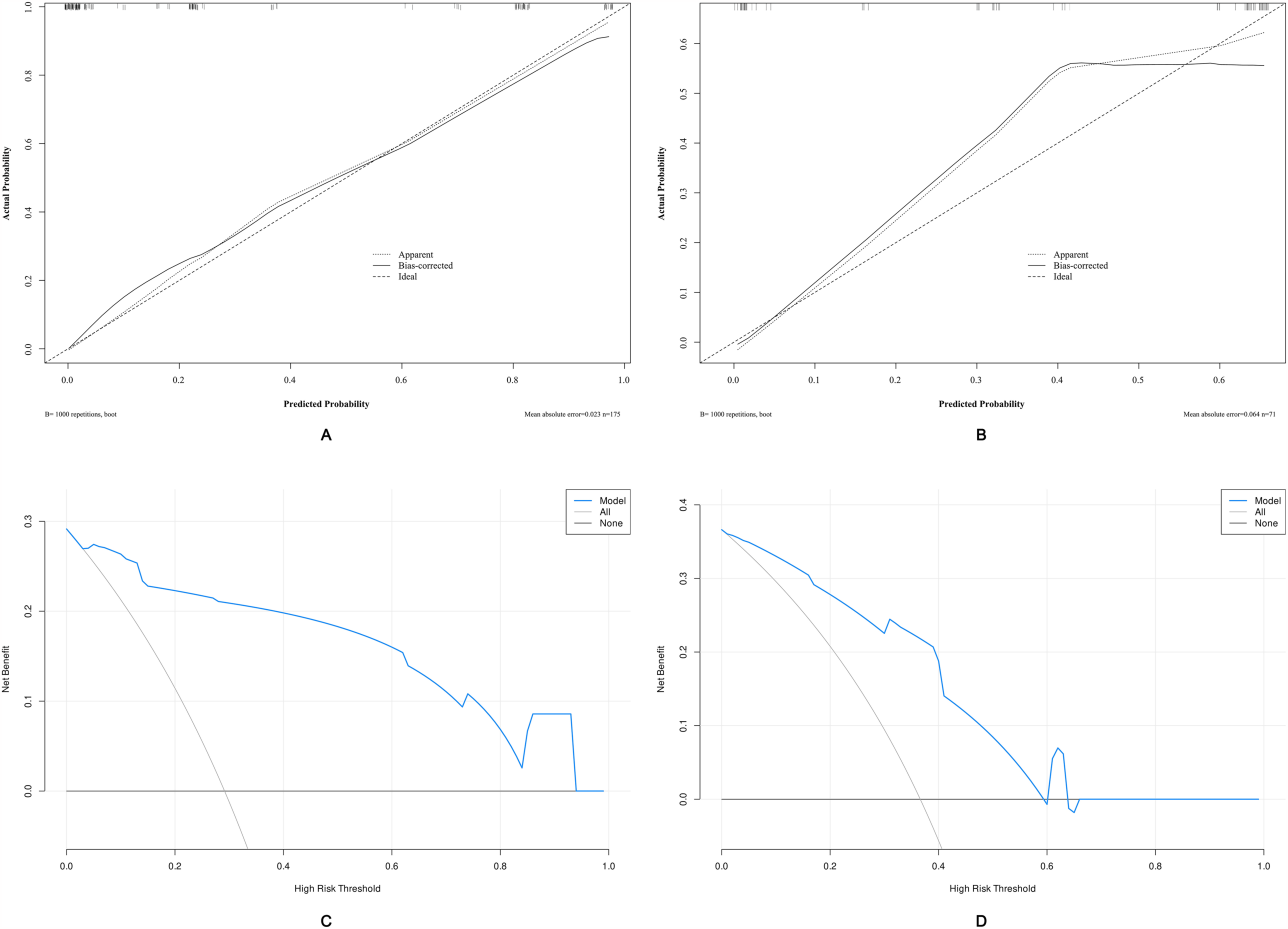
Calibration curves and decision curve analysis (DCA) of the nomogram-based risk prediction model for predicting unfavorable outcomes in patients with PTBI. **(A)** Calibration curve of the nomogram in the development cohort. **(B)** Calibration curve of the nomogram in the external validation cohort. **(C)** DCA for the development cohort. **(D)** DCA for the external validation cohort. The threshold probability and net benefit are represented on the *X*-axis and *Y*-axis, respectively. The blue line indicates the net benefit of the nomogram at different threshold probabilities. The area between the “all-negative” (black line) and “all-positive” (gray line) curves in the DCA plot reflects the clinical utility of the model.

ROC analysis revealed that the AUC for the nomogram model was 0.947 (95% CI = 0.918–0.977) in the development cohort and 0.834 (95% CI = 0.741–0.926) in the external validation cohort (Figure 5). The calibration curves demonstrated that the calibration lines in both the development and external validation cohorts were close to the ideal line, indicating good predictive accuracy (Figures 6A,B). The Hosmer-Lemeshow test yielded *p*-values of 0.944 and 0.593 for the development and external validation cohorts, respectively, further indicating that the model possesses good calibration ability.

### Evaluation of the clinical utility of the nomogram

Applying DCA to evaluate the clinical utility of the nomogram in the development cohort (Figure 6C) and the external validation cohort (Figure 6D) provides insights into the clinical benefits within a reasonable range of threshold probabilities. The DCA results demonstrate that using the nomogram developed in this study to predict unfavorable outcomes in pediatric traumatic brain injury patients offers greater benefits compared to the strategies of treating all patients or treating none.

## Discussion

In this study, we identified the risk factors for unfavorable outcomes in patients with PTBI and developed a predictive scoring scale based on these factors. To the best of our knowledge, this is the first study to develop a scoring scale for unfavorable outcomes in patients with PTBI. Our research demonstrated that a GCS score ≤8, SDH, SAH, and coagulopathy are independent risk factors for unfavorable outcomes in patients with PTBI. When using the nomogram model, the ROC curve, calibration curves, and DCA demonstrated good predictive performance and clinical efficacy. Therefore, this prediction model can be effectively applied in clinical practice for the timely prediction of unfavorable outcomes in PTBI.

The GCS serves as a vital tool for assessing the severity of patient conditions and predicting outcomes in TBI cases because of its simplicity and widespread clinical use. It quantifies various indicators of consciousness and provides crucial insight into the severity of PTBI and patient prognosis (18, 19). However, it is important to note that GCS scores may be influenced by factors beyond TBI (20), and prior studies that included patients with multiple injuries could lead to variations in prognostic assessment (21, 22). In this study, the GCS score at admission emerged as a risk factor for poor prognosis, which is consistent with previous research. Notably, our study exclusively focused on patients with isolated PTBI, striving to minimize the influence of severe injuries to other body parts on the accuracy of GCS assessment upon admission. This underscores the reliability of the GCS score at admission as a risk factor for unfavorable outcomes in patients with PTBI.

Acute SDH is highly prevalent in TBI and serves as a major determinant of short-term outcomes in patients with TBI (23). Lee et al.'s study (23) found that isolated SDH may result in poorer outcomes, compared with other isolated traumatic intracranial hemorrhages, making it a crucial factor in adverse prognosis. Combining known adverse prognostic factors, such as advanced age and low GCS scores, can yield more accurate predictive results. SDH is also associated with the occurrence of traumatic hydrocephalus in children, potentially prolonging hospital stays and increasing healthcare costs, thus contributing to poor prognosis (24, 25). The efficacy of emergency surgery in patients with acute SDH remains debatable. A prospective study by van Essen et al. (26) found that emergency surgery for patients with acute SDH did not offer superior outcomes, compared with conservative treatment, and was not associated with a better prognosis. However, existing research has predominantly focused on adults, with limited studies on pediatric patients. Future multicenter prospective studies are required to ascertain the efficacy of emergency surgery for acute SDH in pediatric patients.

SAH has been associated with increased mortality rates in patients with PTBI and has independent predictive capabilities for in-hospital mortality (27). Chen et al. (27) conducted a prognostic analysis of 550 patients with severe PTBI and found that SAH was associated with in-hospital mortality rates. Oearsakul et al. (28) developed a nomogram for predicting the 6-month prognosis of patients with moderate or severe PTBI, revealing that SAH could serve as an indicator of adverse prognosis. Consistent with previous research, this study demonstrates that SAH is an independent risk factor for adverse prognosis in patients with PTBI. However, in a study by Hochstadter et al. (29), although SAH was found to be associated with unfavorable outcomes in univariate analysis, multivariate analysis failed to demonstrate an independent association between SAH and mortality rates. This discrepancy may stem from the differences in patient populations between this study and that of Hochstadter et al., as all patients in this study were patients with PTBI who remained after excluding those with concomitant extracranial injuries. A study on adult TBI suggested that traumatic SAH could lead to severe vasospasm, the mechanism of which may involve TBI-induced cerebral vascular stretching and bleeding. This leads to the production of spasmogenic and neuroinflammatory substances, causing cerebral vasospasm and further exacerbating brain edema, resulting in an adverse prognosis in TBI patients (30). Although PTBI may also lead to cerebral vasospasm, there is no evidence to suggest that it is specific to SAH (31). Further research is needed to elucidate the potential relationship between SAH and cerebral vasospasm in children.

Although coagulopathy resulting from TBI has long been considered a risk factor for adverse outcomes in adult patients with TBI, little is known about its impact on the prognosis of patients with PTBI (32, 33). There is also a possibility of TBI-induced coagulopathy in children (34). Chong et al. (15) found that patients are prone to early coagulopathy, which is associated with patient mortality and poor functional outcomes. The results of this study emphasize that coagulopathy is an independent risk factor for adverse prognosis in patients with PTBI, suggesting that early monitoring of coagulopathy may help improve the prognosis of patients with PTBI. Additionally, some studies suggest that the early use of tranexamic acid may improve the prognosis of mild-to-moderate adult patients with TBI (35), although its impact on the prognosis of patients with moderate-to-severe TBI is not clear (36). However, there is currently no evidence suggesting that correcting coagulopathy improves the prognosis of patients with PTBI. Future research needs to comprehensively understand the specific impact and potential mechanisms of coagulopathy on the prognosis of patients with PTBI, as well as the effectiveness of relevant treatment strategies.

Interestingly, we did not observe a significant impact of age, skull base fractures, or EDH on PTBI outcomes. Several factors may explain these findings. First, our study included only children aged 16 years or younger, resulting in a narrow age range with limited physiological and metabolic variability; thus, the effect of age on outcomes may not be as pronounced as in adult populations (37). Moreover, the relatively short follow-up period may have precluded the detection of age-related effects on long-term cognitive or neurodevelopmental outcomes. Regarding skull base fractures, although they are typically associated with high-energy trauma, children exhibit greater cranial plasticity than adults, which can buffer against rapid increases in intracranial pressure and mitigate the negative impact of skull base fractures on outcomes (37). As for EDH, which is usually of arterial origin, prompt surgical evacuation can quickly alleviate mass effect and significantly improve outcomes (38). Additionally, our study excluded patients who were declared dead upon admission and were unable to undergo imaging, and these cases might have included severe EDH patients who did not receive timely surgery. This exclusion could have introduced a bias, favoring better outcomes among survivors with EDH. Future studies should refine injury classification, expand the sample size, and incorporate long-term follow-up to validate these findings.

In previous studies (39–41), significant efforts have been made to develop predictive models aimed at identifying unfavorable outcomes in patients with PTBI, with the goal of early identification of high-risk individuals to improve their prognosis. However, most of these studies have primarily focused on clinical variables, with only a few incorporating biochemical and imaging factors (40). For example, Yong et al. (42) adapted models originally developed for adults, such as the IMPACT and CRASH models, for use in pediatric populations. While these adapted models demonstrated reasonable accuracy when applied to children, their performance may still be suboptimal compared to models specifically tailored for pediatric patients (40). Additionally, the study by Caliendo et al. (39) emphasized the critical role of cranial CT findings in predicting outcomes in patients with PTBI. However, their model lacked the inclusion of laboratory indicators, which are essential for a more comprehensive risk assessment. This underscores the need for a simple yet comprehensive prediction model that integrates routine clinical, imaging, and laboratory data.

In our study, we developed a nomogram using readily available clinical, imaging, and laboratory indicators. The model delivered robust predictive performance in both the development and external validation cohorts, underscoring its broader applicability. We propose that integrating this predictive model into the Electronic Medical Record system would allow for a swift assessment of patient risk at admission, providing clinicians with timely prognostic insights. This capability would aid physicians in prioritizing care and efficiently allocating resources, particularly in high-pressure settings like emergency departments or intensive care units. Moreover, the model can enhance communication with patients' families, who often face uncertainty about the prognosis of patients with PTBI. By presenting a clear risk score and outlining potential adverse outcomes, doctors can help families better understand the situation and make informed decisions. This approach not only offers emotional support but also facilitates the development of more personalized treatment plans, especially when critical choices regarding treatment options or invasive procedures must be made.

## Limitations

This study has some limitations. First, as this was a retrospective study, the sample size was relatively small. Additionally, the follow-up period was relatively short, which may not have fully assessed the long-term prognosis of the patients. Hence, further long-term follow-up studies are required. Finally, as this study only included patients from two medical institutions, there may have been regional and institutional biases. Therefore, multi-center collaborative prospective studies are required to validate the external validity and generalizability of our results. Despite these limitations, this study provides important references for the prognostic assessment of patients with PTBI and directions for future research.

## Conclusion

In conclusion, a GCS score ≤8, SDH, SAH, and coagulopathy are independent risk factors for unfavorable Outcomes in PTBI. Based on these findings, we successfully established a risk prediction model that is readily applicable for the prognostic assessment of patients with PTBI, thus improving their prognostic outcomes. Future multicenter prospective studies are warranted to further validate the accuracy of this predictive scoring model, with the aim of enhancing patient outcomes and improving the quality of life.

## Data Availability

The original contributions presented in the study are included in the article/Supplementary Material, further inquiries can be directed to the corresponding authors.
